# The experience of informal caregivers in providing patient care in hospitals in low- and middle-income countries: A qualitative meta-synthesis

**DOI:** 10.1177/13558196221101968

**Published:** 2022-05-19

**Authors:** Unarose Hogan, Amanda Bingley, Hazel Morbey, Catherine Walshe

**Affiliations:** Division of Health Research, Faculty of Health and Medicine, 151268 Lancaster University, Bailrigg, UK

**Keywords:** Low- and middle-income countries, informal caregivers, systematic review

## Abstract

**Objective:**

In low- and middle-income countries, informal caregivers frequently stay in hospitals and perform patient care tasks typically performed by nurses in other contexts. This article reviews qualitative research on these informal caregivers, to gain insight and understanding of their experiences.

**Methods:**

We undertook a qualitative meta-synthesis. Relevant literature was identified through searches of electronic databases in 2021. Thematic analysis was conducted to facilitate the identification of conceptual relationships to formulate synthesised findings.

**Results:**

Twenty-four studies met the inclusion criteria – 13 from Sub-Saharan Africa, five from Bangladesh, two from India, two from Iran, one from Brazil and one from Peru. Three themes were generated from the meta-synthesis: (1) The unwelcome but tolerated guest, (2) Enduring personal sacrifice and (3) Fulfilling familial obligations. These themes emphasised the significant burden associated with the hospital caregiving experience and highlighted the implicit reliance on informal caregivers in low- and middle-income countries.

**Conclusions:**

Informal caregivers perform an essential caregiving role, yet occupy a peripheral and voluntary space in hospitals. There is a clear need to support informal caregivers so that they can safely perform their tasks.

## Introduction

In hospitals in low- and middle-income countries, there is an implicit reliance on informal caregivers to live onsite in the hospital throughout a patient’s stay functioning as ad hoc health care providers.^1–3^ They perform patient care tasks, including feeding, bathing and transporting patients, advocating for patients, administering medicines and preparing meals. This is markedly different from high-income countries in which professional care provision limits and shapes informal caregiver roles in this setting. The practice of informal caregivers taking a central role in patient care more commonly observed in countries with critical shortages of health care workers and may also be attributed to cultural and social norms which deem it more appropriate for a family member to perform intimate patient care.^4,5^

The term ‘informal caregivers’ used throughout this review aligns with the World Health Organization definitions of ‘caregivers’^6(p12)^ and ‘informal assistance’.^6(p34)^ It describes any person without formal health training who is not employed by the hospital facility and is onsite in the capacity as ‘carer’ or ‘guardian’ of a person known to them who is admitted to the facility as a patient. Relying on informal caregivers in low- and middle-income countries to provide patient care more typically delivered by nurses may have implications on the quality of patient care, affecting patient outcomes.

Despite the commonplace presence of informal caregivers in hospitals in low- and middle-income countries, there is no comprehensive overview of evidence of their experience nor clarity on what further research is necessary. Furthermore, available research on informal caregivers is primarily drawn from the quality of life and burden of caregiving surveys or focused on caregiving in the community for chronic diseases and does not distinguish between high-income and low/middle-income countries.^7,8^ The focus of this meta-synthesis was to review qualitative research investigating informal caregivers’ experience of providing patient care in hospitals in low- and middle-income countries to gain insight and understanding of their perspectives.^9^ The specific review question is: ‘How do informal caregivers experience their role in providing patient care in hospitals in low- and middle-income countries?*’*

## Methods

We undertook a qualitative meta-synthesis, following the six steps formulated by Sandelowski and Barroso.^10^ This facilitates interrogation of findings from different studies to produce new interpretations of the data resulting in a more holistic understanding of the experience. This approach is well suited to exploring individual experiences rooted in a constructivist epistemology.^11,12^ The practical value of systematic qualitative synthesis lies in the collation of qualitative material for other researchers who would not have the capacity to locate, read and interpret all the material individually. The protocol for this systematic review is available on PROSPERO (https://www.crd.york.ac.uk/prospero/), number CRD42017082345.

### Step 1: Formulating a review question

A SPIDER model approach was used, covering:^13^

• Sample: informal caregivers in hospitals in low- and middle-income countries
• Phenomenon of Interest: experience perspective, attitudes, views, beliefs and perspectives of informal caregivers engaged in care provision in hospitals in low- and middle-income countries.
• Design: ethnography, phenomenology, grounded theory, all qualitative variations, interviews and observation
• Evaluation: the experience of caregiving
• Research Type: qualitative

### Step 2: Comprehensive systematic search

We obtained studies on the experiences of informal caregivers in hospitals in low- and middle-income countries. Four categories of search terms were used: ‘informal caregivers’, ‘hospitals in low- and middle-income countries list’, ‘patient care’ and ‘personal experience’. Relevant literature was identified by searching the electronic databases MEDLINE, Embase, CINAHL, PubMed and PsycINFO between 5 June and 20 July 2021 inclusive (see online supplement 1).

The process involved iterative searches and hand searching, backward and forward citation searching and berry picking (a non-linear circuitous search process).^14^ Search results were read for relevance and applied to the inclusion and exclusion criteria for review, outlined in online supplement 1. Initial exclusion was performed by the primary author (UH) with consensus on the final list of articles to be examined derived from a discussion with two researchers (AB and CW). This process is depicted in online supplement 1.

### Step 3: Study selection and appraisal

Studies meeting the inclusion criteria were appraised using the Critical Appraisal Skills Programme, which is widely used to demonstrate rigour in the quality appraisal of evidence to be synthesised.^12^ The purpose of this assessment was to ensure the accurate identification of the explicit elements of quality in qualitative research, avoid drawing unreliable conclusions and consider the degree of reflexivity of the researcher as a hallmark for quality.^9,15^ The appraisal was not intended to be a tool for exclusion of studies. That is because research participants' perspectives can be accurately represented even in low-quality studies, and research findings may still be relevant.^16^

### Step 4: Analysing and synthesising findings

A thematic approach was used to synthesise the qualitative studies. Thematic analysis is a means of analysing and reporting patterns identified within and across data sets. It is flexible in process and can provide insight into the experience of individual and factors influencing their actions.^16^

### Step 5: Quality Control of synthesis output

For consistent data to be extracted for this systematic review, a qualitative data extraction tool was adapted from an existing National Institute of Clinical Excellence tool. The domains of study setting, objectives, design, data collection and analysis, methods and themes were extracted from the primary research articles. Given the exploratory nature of this systematic review, ‘findings’ were understood to be ‘key concepts’ arising from the research findings and other relevant details derived from various parts of the publication.^16^ Relevant information included all data related to informal caregivers' experience, perspective, attitudes, views, beliefs and practices regarding patient care provision in hospitals.

Using NVivo software, data were coded (UH) and organised hierarchically into ‘coding trees’ according to emergent concepts and their relationship to one another. The codes generated were not predefined and faced continual refinement, analysis and critical reflection to check their validity. Codes or ‘labels’ attached to the data highlighted points of interest to the review and facilitated the identification of conceptual relationships between codes.^17^

### Step 6: Presenting the findings

Through constant comparison and repeatedly examining data, final themes were isolated across the studies, which were used to formulate synthesised findings. Consensus on each step was reached between UH, AB and CW.

## Results

One hundred and thirty-two papers were assessed for inclusion, and the final number of papers obtained from all combined strategies was 24. The final list of included studies is given in online supplement 2. Studies retrieved were conducted in a variety of geographical locations: 13 from Sub-Saharan Africa (Malawi (3), Kenya (2), Uganda (2), Ghana (1), Niger (1), Tanzania (1), Lesotho (1), Nigeria (1), Mozambique (1)) five from Bangladesh, two from India, two from Iran, one from Brazil and one from Peru. The studies included 689 caregivers in hospitals across these countries.

The findings of the review led to the construction of three overarching themes: (1) The unwelcome but tolerated guest, (2) Enduring personal sacrifice and (3) Fulfilling familial obligations. Each is now discussed in detail.

### Theme 1: The unwelcome but tolerated guest

For many informal caregivers being in the hospital meant feeling marginalised as the hospital made little accommodation for their presence. Caregivers perceived that they were undervalued for their contribution yet tolerated out of necessity for cultural and workload reasons. They felt that being an informal caregiver resulted in their being stereotyped as having little knowledge and ability to care for the patient. They felt their sense of power and autonomy was diminished in the hospital environment.

All 24 articles found that hospital environments were ill-equipped to accommodate the presence of informal caregivers. The caregivers lacked good access to bathrooms, showers, potable water and cooking facilities. Frequently, informal caregivers shared beds with patients or slept on the ground outside the hospital.^5,18–21^ Some informal caregivers reported that hospital conditions deterred patients from seeking timely hospital care. As one informal caregiver in India said: ‘Nobody would stay here unless compelled to’.^21(p89)^

This issue was especially problematic for female caregivers, who were the predominant caregivers in the studies (which is consistent with the cultural norms of caring roles).^5,22–24^

The gender issues involved in informal caregiving are discussed further below.

The hospital environment became a space where domestic and medical boundaries overlapped, due to the often extended nature of informal caregivers’ stays in the hospital. As one caregiver reported:

All around were signs of home, family, and faith. Family members frequently outnumbered patients on the wards...The trappings of home surrounded hospital beds: food, plastic basins, blankets, clothes, and bibles.^22(p19)^

This disorder contributed to caregivers’ concerns that patients were living in unhygienic conditions.^5,21,25^ Bathrooms were often reported to be contaminated with excrement and generally dirty, even when individuals actually paid to use them.^20,21,25^

The caregivers reported that hygiene supplies were largely absent on the wards, and materials such as gloves and cotton wool were not reliably available.^3,5,22^ They had little access to soap for handwashing, even when cleaning up a patient’s urine and felt hampered in providing quality patient care due to these limitations.^26^ They often used their own clothes and cloths to clean patients.^3^

The close physical proximity of patients and caregivers risked disease transmission.^3,7,8^ One study reported informal caregivers had some, albeit limited, understanding of disease transmission, describing direct contact with contaminated surfaces and waterborne disease as major modes of transmission.^3^ This limited understanding was exemplified by a caregiver in Kenya refusing to wear gloves when cleaning blood: ‘She is my daughter, what can she give me?’^21(p22)^ However, no research reported an informal caregiver having been infected with a hospital-acquired infection through their activities.^7^

The literature identified an implicit reliance on informal caregivers to provide essential patient care.^3,7,27^An informal caregiver in a Ghanaian study noted: ‘Some of the basic things that nurses should do, they are looking up to the patient’s family to do it’.^28(p7)^ Yet hospital staff would sometimes protest their presence. One health care worker said, ‘informal caregivers interfere in nursing activities; some of them have incomplete information and want to use it.’^8(p883)^ Another complained vociferously:

After a hundred relatives have left the ward, the floor looks like a wasteland. It is not surprising, therefore that the ward cleaners always fight with them. While sweeping up the banana peels, nutshells, and empty chip bags, a cleaner told me, ‘See what these barbars (barbarians) have done. If you go to the toilet, you will see what they have done there, these stupid attendants of the patients come from the village and do not know how to use a toilet.’^29(p283)^

Personal and intimate care of patients was viewed in some social contexts as degrading to nurses.^4^ In some Islamic cultures, physical contact between females and non-family members is prohibited, which interfered with health care workers’ willingness to engage in patient interaction.^3,4^ Implied here is a reliance on informal caregivers to undertake nurse functions that would otherwise go unperformed due to cultural norms. In Bangladesh, for instance, nurses working night duty carry a cultural stigma of being associated with nighttime commercial sex work, which reduces nurses’ ‘bride market’ value.^3^ To reduce the stigma associated with the nursing profession, nurses distance themselves from patients, and patients’ relatives become nurse surrogates.^4,7^ One informal caregiver said:

I think nurses feel they are superior to poor patients and their carers. They don’t like to associate with us because we are poor and dirty. They maintain a distance from these jobs in case their white dress might become dirty.^4(p1171)^

Informal caregivers who felt obliged to perform personal care tasks reported a similar culturally rooted sense of stigma and shame in their role. One caregiver in Nigeria explained that some villagers may become violent given what the caregiver is doing for the caregiver’s mother:

In my culture, children are forbidden from seeing the nakedness of their parents. I am sad when I see her exposed body every day. I always remember that it is unacceptable in our culture to see a parent’s nakedness. But if I do not provide care for her, who else will? I am afraid for my life because of the repercussions of the taboo on me.^30(p2629)^

The burden of caregiving extended to marital and familial relationships, placing strain on these, with several studies reporting worry and fear about the wellbeing of family at home.^2,24,31,32^

As one caregiver reported, ‘Sometimes it brings the morale of the family down, especially with the children.’^31(p321)^

In several studies, informal caregivers perceived health care workers as strict, uncaring and unwilling to do routine patient care. The caregivers rationalised their role as de facto nurses, performing functions nurses were unwilling to do.^3,4^ As one caregiver reported:

They [nurses] think as an educated person these tasks should not be their work, nor should they serve or give personal care to uneducated or poorly educated people.^4(p1171)^

Informal caregivers were frustrated as they felt health care workers did not recognise their contribution to patient care.^18,23,31^ The caregivers expressed a desire for a family-centred approach to care, where they would be active participants in decisions, but felt unable to raise this with health workers, who often stereotyped them as incompetent.^24^ Some informal caregivers felt reluctant to ask the nurses and doctors questions because they were afraid it would result in a reprimand. As noted in one Malawian study:

It is difficult for us to explain the patient's problem to the nurses; for example, you can tell the nurse the patient's problem, and she will answer, 'you do not listen! Wait for the doctor and tell him', this discourages us to ask anything.^20(p808)^

This fear of reprimand was echoed in other studies where informal caregivers had concerns over patient care, such as worrying that drawing blood frequently would weaken their child, especially when they did not understand why it was necessary.^24,29^

In general, informal caregivers were more likely to criticise nurses’, rather than doctors’, attitudes towards them. This may be indicative of the fact caregivers interact with nurses with more frequently and that doctors have a higher perceived status. The sense of disempowerment felt by informal caregivers and their subservience to the authority of health workers within the hospital environment featured prominently.^24,27,33^ Informal caregivers frequently expressed a desire to feel less marginalised. As one daughter reported:

I just sit here like a robot. Nurses asked me to buy items that my mother needed. They never told me why she needed them. They ordered me to pay for dialysis, laboratory investigations and other things. I don’t like it when I do not know the reason behind my actions. I am sad to see myself as a fool being tossed around.^30(p2627)^

Informal caregivers felt they added value to patient care, but that this was unrecognised by health workers, who saw them as poor and uneducated.^7,31^

### Theme 2: Enduring personal sacrifice

For many informal caregivers being in the hospital imposed significant personal sacrifice. Their caregiving role in the hospital negatively impacted their physical, emotional and social wellbeing. The impact of the caregiving experience was not confined within the hospital boundaries; rather, relationships and job prospects outside the hospital also suffered. The caregiving role also carried the burden of various costs.

Informal caregivers experienced physical injuries associated with caregiving, including hip pain and back problems from lifting patients.^34^ They faced barriers in accessing nutrition and caring for their personal health when in the hospital.^21^ Caregivers described day-to-day care as exhausting and stressful. They frequently experienced fatigue and insomnia, which was particularly pronounced among older caregivers.^18,31,35,36^ Integral to the caregivers’ emotions was the physical state of the patient they were looking after, the patient’s deteriorating health and fear of death.^28,37,38^

Prolonged periods of caregiving saw some caregivers experience depressive symptoms such as tiredness, sadness, difficulty sleeping, lack of motivation and suicidal ideation.^30,34,36^ Informal caregivers worried that they exacerbated the patient’s discomfort, confirmed by a patient’s body language or complaints.^37,38^ For instance, caregivers caring for HIV and AIDS patients felt discomfort and repulsion when dealing with the patients’ bodily fluids, and feared the patients would misinterpret this as an unwillingness to help.^37^ They also were stressed by the uncertainty over a patient’s condition, the patient’s physical condition and unknown HIV status.^37^ One caregiver said:

I was using those Dettols [disinfectants], but I am worried that I could be having it [HIV] already. Maybe he has infected me. I am worried that I have it because I did not protect myself when I was touching all that stuff and bathing him…no one gave me the gloves, not even the nurse at the hospital.^37(p23)^

The death of a loved one were traumatic for some informal caregivers and contributed to their emotional burden.^23,36^

Caregivers described how caregiving had imposed restrictions on their lives and prevented them from engaging in social activities.^30^ Stressors included a lack of independence and time to engage in social activities due to their having to be at the hospital.^34^ Social isolation included loneliness, conflicts with other family members regarding decisions, lack of support from the broader family, being unable to undertake other regular social activities (such as playing with their children) and having to put their social life on hold.^23,24,31,34^

Often mentioned was the impact on the informal caregiver’s ability to earn a livelihood. Most caregivers stopped working altogether in order to care for their family members. As one explained:

I abandoned my search for [a] job because I have to provide care. I felt that my future plan has been jeopardised since it is impossible for me to apply for a job from the hospital environment. My colleagues are employed and making future plans while I am here providing care.^30(p2627)^

Informal caregivers faced difficulties in balancing their professional, family and caregiving roles, as they were unable to be physically present in all places.^21,31,32^ Several mothers reported concerns of being unavailable to care for other children at home while others brought young infants to the hospital to remain with them.^21,31,32^ Some informal caregivers began looking after the children of the sick patient, extending their role beyond the hospital. One Ugandan caregiver providing care for her widowed HIV-positive sister reported she cared for 20 children at home, including her sister’s children.^31^ Caregiving had impacts on marital relationships, with several studies reporting worry and fear about the wellbeing of spouses at home.^21,31,32^

Informal caregivers bore significant financial burdens due to caregiving.^23^ Being unable to work, many were wholly dependent on the financial support of other family members (who sometimes failed to do so consistently). Caregivers assumed responsibility for the expenses associated with care, including the cost of drugs, food, transport to the hospital and other associated costs. Informal caregivers mostly relied on collective financial support from family networks to meet these costs.^18,20,31,34^ In Nigeria, one informal caregiver noted:

I sleep outside on the veranda within the hospital with other family caregivers…I couldn’t afford the hostel fee; if I pay the hostel fee, it may affect our ability to buy drugs, so I sleep outside.^28(p6)^

### Theme 3: Fulfilling familial obligations

For many informal caregivers being in the hospital meant they were fulfilling what they regarded as an inevitable role and duty. In some cases, this made it easier for caregivers to adapt to the caregiving role. It also fostered closer relationships and empathy for their patients. But in other cases, it brought an additional strain to the relationship.

Most studies identified familial obligation as the reason a caregiver took on the caregiving role.^30,36^ A healthy caregiver–patient relationships strengthened the informal caregiver’s ability to fulfil their role.^31,36^ Time the caregiver spent with the patient promoted a closer relationship between them, particularly when caregiving was prolonged.^30^ There was a sense of pride among informal caregivers at being able to perform a caregiving role for their family member, strengthening the relationship. As a wife caring for her husband commented:

I feel fulfilled that I am able to provide care to my husband...of 30 years...The type of care I provide for him makes him happy, and it has strengthened our love for each other.^30(p2629)^

Caregivers empathised with their patients, notably when the patient was in pain. Empathy was particularly pronounced in a maternal-child relationship. As one 71-year-old woman caring for her son said: ‘I’d rather die just to save him.’^31(p321)^

When patients were resistant to receiving care, the informal caregiver-patient relationship suffered. Informal caregivers described some patients as ‘difficult’,^34(p36)^ ‘demanding’^34(p36)^ and ‘bad-tempered’^34(p36)^ and noted they refused medication or personal care. Informal caregivers who experienced this felt anger, frustration and stressed.^37^

Women primarily fulfilled the caregiving role. Males typically only undertook caregiving when it would impractical for the women to do so (for instance, the women had to look after young children or were physically incapable of doing caregiving work).^22,29,32^ But a Bangladeshi study found the male informal caregivers cared for male patients. This is because it would be inappropriate for women to stay in a male hospital ward as that would breach the practice of purdah (seclusion of females from males and strangers).^29^

In some cases, fulfilling this familial duty led caregivers to accept the role with a sense of positive wellbeing.^24,27,29,31^ Informal caregivers were positively influenced by previous caregiving experience.^28^ Gaining skills as a caregiver led to enhanced feelings of confidence, further increasing the ease with which they adapted to the role.^7,21^ Closely linked to confidence were feelings of resilience brought about by the acquisition of new skills, as described by one informal caregiver in India:

Baba (father) always took all major decisions, related to managing finances, property, major family decision. Now that he is not well, I am learning to manage those.^21(p88)^

Coping strategies which supported ease of adapting to the caregiving role included a positive attitude to the situation, achieved through good humour and avoiding seeing caretaking as an obligation.^28,36^ One caregiver explained that sharing caregiving responsibilities helped her have a more autonomous life, reducing the emotional burden of caregiving.^34^ Informal caregivers used religion, spirituality, prayer and meditation as sources of strength and resilience to manage anger and anxiety.^31,34,39^

Informal caregivers came to rely on other informal caregivers as a source of support. They developed a social bond at the hospital and took responsibility to notify each other in case of an emergency, ensuring that the absent caregiver had time for rest.^21^ According to one Bangladeshi informal caregiver:

We (family caregivers) stay here (in-patient ward) like a family. If one family caregiver has to go to call the nurse and leaves their patient alone, I stay near that patient and keep an eye on him or her.^3(p308)^

## Discussion

Our findings provide a consistent account of the distinctive experience of informal caregivers providing care to family members in hospitals in low- and middle-income countries.

The first theme, the unwelcome but tolerated guest, highlights the fact that informal caregivers are an essential, albeit frequently unrecognised, group of care providers in hospitals in low- and middle-income countries. Underpinning this is the lack of power the informal caregiver has in the hospital context, with little influence over decision-making in patient care.^40^

There are considerable advantages in involving family members in patient care, and these are associated with a range of positive outcomes, including reducing the stress of providing such care, improving patient empowerment and improving relationships with health care workers.^41^ But informal caregivers perceived health care workers as apathetic towards their caregiving contributions. Hospitals should support and upskill informal caregivers. The unskilled provision of patient care is consistently associated with poorer patient outcomes, and the need for improved health literacy is most acute in low- and middle-income countries.^42^

Adding informal caregivers to patients’ care team may open up access to better resources, protections and education for the health system.^35,43^ Legislation has been suggested in Cambodia to cover how nurses should supervise unqualified service providers.^44^ Similar laws could be considered elsewhere.

The second theme, enduring personal sacrifice, emphasises the stressful burden of undertaking informal caregiving.^41,45^ This is consistent with the theoretical framework of the Stress Process Model, which identifies caregiver stress as a complex response to internal and external demands affecting psychological, relational and social resources interactions.^36^ While the burden of caregiving is a universal experience, its effects may be most acutely felt when considered against in a context of poverty and other pre-existing stressors. The need to develop and integrate psychosocial interventions – such as peer support, and stigma- and anxiety-reduction programmes – is vital to support caregivers to continue their role.^45^

The direct and indirect financial costs associated with caregiving are likely to have a more pronounced impact in low- and middle-income countries.^7,31^ Moreover, continued future reliance on the informal caregiver group to provide patient care cannot be assured in modern contexts where urban migration for employment and women working outside the home may reduce the future availability of informal caregivers.^31,45,46^ Financial support for informal caregivers – including to cover accommodation, meals and transport – could be considered.^41^

The third theme, fulfilling familial obligations, reflects the sense of duty among caregivers and their ability to adapt to their role. Indeed, caregivers can find the role rewarding and life enhancing.^23,30^ Caregivers seek out family support, forge alliances with other caregivers and use spiritual practices as strategies to cope with the role.^31,47^ To maintain a good quality relationship between the caregiver and patient, support programmes have been effective in reducing caregiver distress and fostering higher self-esteem among caregivers.^49^

Informal caregivers are frequently women. This potentially exacerbates an enduring gender inequality in low- and middle-income countries by perpetuating a system that prevents women entering paid employment and, instead, leaves them in poverty.^48^ Gendered relations of caregiving need to be addressed in policies and practices for caregiving to reduce the impact on women and account for the responsibilities of traditional roles of women in many of the countries.^48^

One gap in the literature is the lack of research into informal caregivers’ potential exposure to disease. The occupational risk of disease transmission among health care workers is well established, but a similar risk may exist among informal caregivers who perform patient care.^49^ The patient care practices caregivers perform, their exposure to the hospital environment and lack of access to infection prevention and control infrastructure, as well as their limited understanding of disease transmission and preventative practices, may leave caregivers exposed to greater risk of infection. No study in this review reported whether an informal caregiver was exposed to or transmitted an infectious agent while in a hospital. Indeed, low- and middle-income countries frequently lack the necessary laboratory support and hospital-acquired infection surveillance systems necessary for hospital-associated infection reporting.^49^ Several studies elsewhere have acknowledged the role of family members as contributors to hospital transmission of disease.^50^ In today’s context of the COVID-19 pandemic and emerging pathogens, adherence to best infection prevention and control principles by all people in hospitals is essential. This must include informal caregivers.

This review did not explore the perception of health care workers towards informal caregivers, how that may influence the development of a better therapeutic relationship to support the patient and how willing health care workers would be to being informal caregiver educators. This is an area for further research to inform appropriate educational and supportive interventions.

## Limitations

There are four main limitations to this study. First, although the included studies had a broad geographical range, many countries where informal caregivers are typical were underrepresented or did not appear at all.

Second, our study was obliged to focus on interview-based research rather than observation. That is because the majority of the included studies used in-depth interviews or focus groups as their data collection method. Only eight studies adopted observation as a component of their data collection.^3,18,21,22,26,27,29,33^ There can be differences between what interviewees self-report and what an independent researcher observes. This indicates a need for additional observational qualitative research to further understand the experience of the informal caregiver.

Third, the rigorous Critical Appraisal Skills Programme tool used for analysing the included studies can only assess the reporting of these studies, rather than how they were conducted. Our study therefore required a subjective judgement that the studies did accurately reflect the perspectives of the research participants.^12^

Fourth, our study did not include any research on caregiver restrictions that may have been imposed due to the COVID-19 pandemic. The importance of the informal caregiver role as a potential bridging population between the hospital and the community in view of COVID-19 and other forms of infection warrants further research.

## Conclusions

Informal caregivers perform an essential caregiving role in hospitals in low- and middle-income countries but occupy a peripheral and voluntary space in the hospitals. In countries where a critical shortage of health care workers and a cultural reliance on informal caregivers persists, continuing to devalue them within the health system may be detrimental to patient outcomes and negatively affect the broader population health and health system. There is a clear need to support informal caregivers so that they can perform their role safely.

## Supplemental Material

Supplemental Material - The experience of informal caregivers in providing patient care in hospitals in low- and middle-income countries: A qualitative meta-synthesis Click here for additional data file. Supplemental Material for The experience of informal caregivers in providing patient care in hospitals in low- and middle-income countries: A qualitative meta-synthesis by Unarose Hogan, Amanda Bingley, Hazel Morbey and Catherine Walshe in Journal of Health Services Research & Policy
