# Supplementary material for: The experience of informal caregivers in providing patient care in hospitals in low- and middle-income countries: A qualitative meta-synthesis
Source: J Health Serv Res Policy. 2022 May 19;27(4):321–9. doi: 10.1177/13558196221101968 (PMC9548935; doi:10.1177/13558196221101968)
Supplement: Supplemental Material - The experience of informal caregivers in providing patient care in hospitals in low- and middle-income countries: A qualitative meta-synthesis [file sj-pdf-1-hsr-10.1177_13558196221101968.pdf]

Journal of Health Services Research & Policy
The experience of informal caregivers in providing patient care in hospitals in low- and middle-income countries: A qualitative meta-synthesis
Hogan U, Bingley A, Morbey H and Walshe C

SUPPLEMENT 1

Table S1: Database sources

Data Source	Date of Search	Rationale
**Databases**		
**CINAHL**	**05/06/2021**	Nursing care related to patient care provision
**EMBASE**	**29/06/2021**	Biomedical database review for related literature
**MEDLINE**	**05/06/2021-20/06/2021**	Check for existing published literature relate to central topics
**PROSPERO**	**05/06/2021**	Check for existing systematic reviews in related topics
**PsycINFO**	**06/06/2021**	Check around a concept for the emotional burden of caregiving

Table S2. Inclusion/ Exclusion Criteria

Parameters	Inclusion criteria	Exclusion criteria
**Location**	Low and middle-income countries as defined by the World Bank list	Non-low and middle-income countries count[illegible]
**Setting**	Hospitals in low and middle-income countries	Community or non-acute patient care setting
**Language**	Studies written in English	Non-English Language studies
**Time frame**	All available	None
**Population**	Studies that focus on Informal caregivers<br><br>Substantive component of findings related to informal caregivers	Studies focused exclusively on healthcare workers in low and middle-income countries<br><br>Studies focused on home-based care<br><br>Community-based Informal caregivers
**Outcome**	Studies concerned with practices of informal caregivers in providing patient care. Experiences, perspectives, attitudes, views, and beliefs of ICs in providing patient care	None
**Study Type**	Qualitative Primary Research/ Systematic Reviews	Quantitative research
	Studies that report on qualitative, mixed-methods	Books, opinion pieces, policy documents

Journal of Health Services Research & Policy
The experience of informal caregivers in providing patient care in hospitals in low- and middle-income countries: A qualitative meta-synthesis
Hogan U, Bingley A, Morbey H and Walshe C

Figure S1 PRISMA Flow Diagram

SUPPLEMENT 2

Table S1: Studies Included in Review

Author/ Year<br><br>Country	Aim	Qualitative approach	Participants	Data collection	Analysis approach	Finding
**Aziato et al. 2014**<br><br>**Ghana**	Explore psychosocial factors influencing caregivers during the care of surgical patients	Exploratory qualitative design	12 Caregivers<br><br>1 Key informant	Interviews	Thematic validation	faith, fe<br>commit<br>knowle<br>caregiv
**Amiresmaili (2018)**<br><br>**Iran**	Show opportunities and threats of informal carers in hospital	Phenomenol ogy	22 Caregivers<br><br>9 Nurses	Semi-structured interviews	Thematic Analysis	Caregiv<br>patient<br>include<br>system<br>so
**Bhattacharyya & Chatterjee (2019)**<br><br>**India**	Explore the experience of family caregivers of hospitalised older people in Kolkata, India	Qualitative – not specified	54 Caregivers	Interviews and field observations	Thematic Analysis	For car<br>enviror<br>impacte<br>financia
**Brown et al. 2011**<br><br>**Kenya**	Explore the relationship between the (medical) hospital and (domestic) spaces and how the home is (re)made and inverted within the hospital wards caregivers	Ethnograph y	Not specified	Observation	Not Specified	The or<br>within<br>patients<br>diverge<br>medical
**d'Alessandro (2015).**<br><br>**Niger**	To describe practices and issues of medical and personal care and hospital hygiene and compare to IPC standards	Anthropolog y	8 caregivers<br><br>56 nurse, doctors and cleaners	Interviews and field observations	Thematic Analysis	Persona<br>perforn<br>family<br>charact<br>unhygie
**Eslami et al. 2018**<br><br>**Iran**	To explain the perspectives and experiences among caregivers of the patients undergoing hemodialysis in Iran	A descriptive exploratory qualitative design	25 family caregivers	Interviews and field observations	Thematic Analysis	Caregiv<br>such as<br>care, t<br>emotion
**Grant 2003**	Discuss patient and caregiver end of life	Qualitative - not	32 patients and their	Interview	Thematic	Caregiv<br>role in

**Kenya**	experiences among those with cancer or AIDS	specified	caregivers		Analysis	emotior and fin patients
**Hadley et al. (2007)**<br>**Bangladesh**	Factors that influence nurses' behaviour in the provision of 'hands-on' care in hospitals nurses perception	Mixed Methods	18 key informants- Caregivers, Health Workers & Patients	SS Interviews<br>Focus Groups	Thematic Analysis	Caregiv surroga patient were ur
**Hoffman et al. (2014)**<br>**Malawi**	To characterise the caregiver population and explore their role in the health system of a Hospital	Mixed Methods	73 participants, 60 caregivers, 13 hospital staff	Interviews, Semi Qualitative Survey	Thematic Analysis	Caregiv range c had litt in the conflict
**Islam et al. (2014)**<br>**Bangladesh**	Explore family caregivers' perceptions and practices related to disease transmission in hospitals.	Ethnographi c	12 caregivers	Observation	Thematic Analysis	Caregiv multipl without infectio and are disease
**Israel et al. 2008**<br>**Malawi**	Gain insight into the guardians' perspective on cancer treatment, especially concerning factors which could influence abandonment of treatment	Qualitative – not specified	25 caregivers	Interviews, Focus Groups	Grounded framework approach	Caregiv concerr diagnos impose financia
**Jagannathan et. al 2011**<br>**India**	To explore the needs of caregivers of in-patients with schizophrenia in India.	Qualitative – not specified	30 caregivers	Focus Groups	Thematic Analysis	Caregiv unmet educati schizop health
**Makoae, M.G. (2009)**<br>**Lesotho**	Caregivers' experiences with the bodily care of AIDS patients before antiretroviral therapies were available	Phenomenol ogy	21 caregivers	Interviews	Phenomenol ogical approach	Caregiv sympat perforn
**Mwangi et al. (2008).**<br>**Tanzania**	Experience and perceptions of paediatric in-patient care among mother caregivers	Qualitative – not specified	261 mother caregivers	Interviews	Thematic Analysis	Mother experie environ unsupp with he

**Oyegbile, Y.O., Brysiewicz, P. (2017)**<br><br>**Nigeria**	Experiences of family caregivers providing care for patients living with End-Stage Renal Disease in Nigeria	Qualitative descriptive study	15 caregivers	Interviews	Thematic Analysis	Caregiv on car diagnos Renal D
**Olwit et al. (2015).**<br><br>**Uganda**	Explore chronic sorrow as an expression of grief as seen among the caregivers of patients diagnosed with schizophrenia in Butabika Mental Hospital in Uganda	Descriptive qualitative design	22 caregivers	Interviews and Focus Groups	Content Analysis	Caregiv sorrow, from th various mostly ones
**Paget et al. (2016).**<br><br>**Malawi**	Explore the perspectives and experiences of caregivers of children with disabilities (CWD) from acquired brain injury in hospital settings	Exploratory Qualitative	6 caregivers from in-patient setting	Interviews, Focus Groups	Thematic Analysis	Caregiv neurodi burden costs, s barriers
**Park et al. 2020**<br><br>**Bangladesh/ Indonesia**<br><br>**(S. Korea excluded)**	Examine whether the role of patients' families has been accounted for in the infection prevention and control (IPC) guidelines and policy	Systematic Review	92 articles included	Systematic search	Thematic Analysis not specified	health primary comes acute ho only ac the 92 a
**Pesantes et al. (2017)**<br><br>**Peru**	Characterise the impact of post-stroke care among Informal caregivers in a hospital setting	Qualitative- not specified	12 caregivers	Interviews	Thematic Analysis	Particip emotion sympto hardshi caregiv
**Söderbäck, M., & Christensson, K. (2007).**<br><br>**Mozambique**	Describe nurses' beliefs and practice regarding family involvement in the care of hospitalised children in Mozambique	Ethnographic	36 nurses	Interviews and field observations	Qualitative content analysis	Nurses caregiv patient shortag their re protect identifi
**Streid et al. 2014**<br><br>**Uganda, South Africa**	Expand understanding of caregiver burden in Africa Caregivers' primary stressors related to day-to-day patient care	Qualitative- not specified	37 caregivers	Interviews *Stress Process Model	Thematic Analysis	Caregiv identifi care, social i respons

**Urizzi & Corrêa 2007**<br><br>**Brazil**	Experience concerning family members of patients in the Intensive Care Unit (ICU)	Phenomenology	17 caregivers	Family interviews	Thematic Analysis	The e[illegible] relative[illegible] patients[illegible] negativ[illegible] family a[illegible]
**Zaman (2004).**<br><br>**Bangladesh**	Hospital culture in the context of Bangladeshi society, Family members, are engaged in nursing and provide various kinds of support to their hospitalised relatives	Ethnography	Not specified	Observation and interviews	Descriptive – not specified	Caregiv[illegible] care to [illegible] to ward[illegible] necessi[illegible]
**Zaman, S. (2013).**<br><br>**Bangladesh**	Explores the experiences of family members who have kin in the hospital	Ethnography	Not specified	Observation and interviews	Descriptive – not specified	Family [illegible] integral [illegible] organis[illegible] Banglad[illegible] perform[illegible]

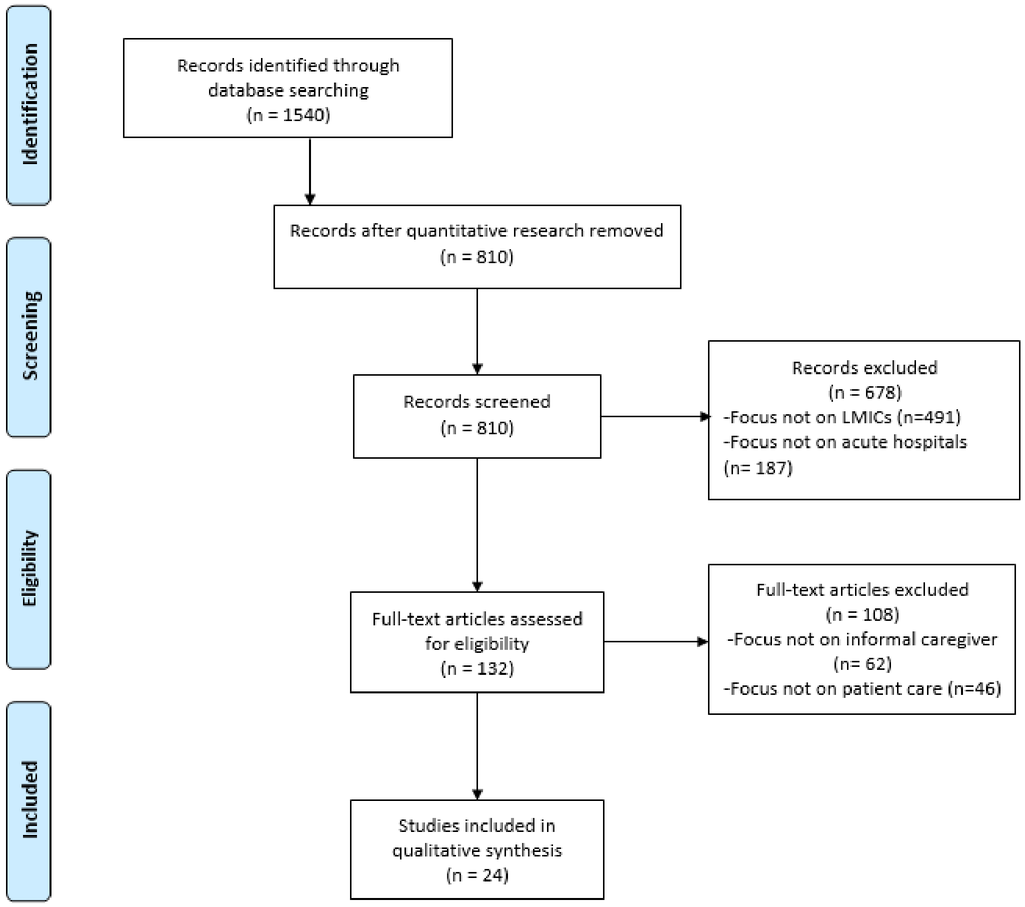
Identification
Screening
Eligibility
Included
Records identified through database searching (n = 1540)
Records after quantitative research removed (n = 810)
Records screened (n = 810)
Records excluded (n = 678) -Focus not on LMICs (n=491) -Focus not on acute hospitals (n= 187)
Full-text articles assessed for eligibility (n = 132)
Full-text articles excluded (n = 108) -Focus not on informal caregiver (n= 62) -Focus not on patient care (n=46)
Studies included in qualitative synthesis (n = 24)

Journal of Health Services Research & Policy

The experience of informal caregivers in providing patient care in hospitals in low- and middle-income countries: A qualitative meta-synthesis

Hogan U, Bingley A, Morbey H and Walshe C
